# Combined Effects of Soy Isoflavones and β-Carotene on Osteoblast Differentiation

**DOI:** 10.3390/ijerph121113750

**Published:** 2015-10-28

**Authors:** Yoriko Nishide, Yuko Tousen, Miki Tadaishi, Masaki Inada, Chisato Miyaura, Marlena C. Kruger, Yoshiko Ishimi

**Affiliations:** 1 Department of Food Function and Labeling, National Institute of Health and Nutrition, National Institutes of Biomedical Innovation, Health and Nutrition, 1-23-1 Toyama, Shinjuku-ku, Tokyo 162-8636, Japan; E-Mails: nishide@nih.go.jp (Y.N.); tousen@nih.go.jp (Y.T.); mt205315@nodai.ac.jp (M.T.); 2 Department of Biotechnology and Life Science, Tokyo University of Agriculture and Technology, 2-24-16 Nakamachi, Koganei, Tokyo 184-8588, Japan; E-Mails: inada@cc.tuat.ac.jp (M.I.); miyaura@cc.tuat.ac.jp (C.M.); 3 Department of Nutritional Science, Faculty of Applied Bioscience, Tokyo University of Agriculture, 1-1-1 Sakuragaoka, Setagaya-ku, Tokyo 156-8502, Japan; 4 School of Food and Nutrition, Massey Institute of Food Science and Technology, Massey University, Private Bag 11-222, Palmerston North 4442, New Zealand; E-Mail: M.C.kruger@massey.ac.nz

**Keywords:** soy isoflavones, β-carotene, osteoblast differentiation, bone

## Abstract

Soy isoflavones, genistein, daidzein and its metabolite equol, as well as β-carotene have been reported to be effective for maintaining bone health. However, it remains to be elucidated whether combining soy isoflavones with β-carotene is beneficial to bone formation. This study investigated the combined effect of soy isoflavones and β-carotene on the differentiation of MC3T3-E1 preosteoblastic cells. Daidzein and genistein alone did not affect cell growth but increased alkaline phosphatase (ALP) activity. Beta-carotene alone inhibited cell growth and markedly enhanced ALP activity. Soy isoflavones combined with β-carotene resulted in higher ALP activity than treatment with isoflavones or β-carotene alone. We observed significant main effects of β-carotene on the enhanced expression of Runx2, ALP, and ostepontin mRNA, whereas there was a significant main effect of soy isoflavones on the expression of osterix mRNA. To investigate how β-carotene affected osteoblast differentiation, MC3T3-E1 cells were treated with retinoic acid receptor (RAR) pan-antagonist combined with β-carotene. Osteopontin and ALP mRNA expression levels, which were increased following treatment with β-carotene, were significantly suppressed by the RAR pan-antagonist. This suggests treatment with β-carotene enhanced early osteoblastic differentiation, at least in part via RAR signaling. These results indicate that a combination of isoflavones and β-carotene may be useful for maintaining a positive balance of bone turnover by inducing osteoblast differentiation.

## 1. Introduction

Functional foods and their bioactive components have received much attention in recent years for their proposed health benefits and potential risk reduction of certain diseases, including osteoporosis. Bone mass is influenced by a number of factors, such as genetics, nutrition, hormonal conditions, exercise, and lifestyle. Bone loss associated with age is caused by decreased bone formation and increased bone resorption, resulting in a significant risk for bone fracture, thus impacting sustaining a healthy lifestyle. Functional foods and their bioactive components may therefore be useful for preventing age-related bone loss and maintaining bone formation.

Soy isoflavones, genistein, daidzein and its metabolite equol, have structural similarities to 17β-estradiol, exhibiting weakly estrogenic action by binding to estrogen receptors (ERs). Soy isoflavone supplementation is reported to decrease the risk of osteoporosis in postmenopausal women [1,2]. Additionally, a number of studies have reported that soy isoflavones dose-dependently inhibit bone loss in both female and male osteoporotic animal models [3]. *In vitro*, soy isoflavones have been shown to suppress osteoclast formation and promote osteoblast differentiation [4,5,6,7]. Thus, soy isoflavones might be major functional food components with the potential to maintain bone health.

On the other hand, β-carotene is the most abundant provitamin A in the human diet, accumulating in plasma and tissues as a parent compound [8]. The metabolites of β-carotene, which act as ligands for the retinoic acid receptor (RAR) family of transcription factors, modulate chronic disease risk and prevent vitamin A deficiency [9]. Epidemiological reports suggest that β-carotene is beneficial for reducing bone loss in older adults [10,11]. Furthermore, several experimental studies report that β-carotene and other carotenoids enhance osteoblastogenesis and suppress osteoclastogenesis [12,13,14]. However, the *in vitro* effects of β-carotene on bone turnover remain to be fully understood. 

Functional foods or their components are often taken in various combinations. However, the efficacy and safety of the combined intake of several types of functional foods or their components have not been adequately addressed. Previously, we reported that a combination of soy isoflavones with carotenoids enhanced the suppressive effect on osteoclastogenesis [14]. Hence, the purpose of this study was to investigate the combined effect of soy isoflavones and β-carotene on osteoblast differentiation *in vitro*.

## 2. Experimental Section

### 2.1. Materials

The clonal mouse MC3T3-E1 preosteoblastic cell line was obtained from Riken BioResource Center (Tsukuba, Japan). Beta-carotene was purchased from Sigma Aldrich (St. Louis, MO, USA) and dissolved in tetrahydrofuran (THF) (Sigma Aldrich). Genistein and daidzein were obtained from Nagara Science (Gifu, Japan); (*R*,*S*)-equol was purchased from LC Laboratories (Woburn, MA, USA). LE540 was obtained from Wako Chemicals (Osaka, Japan). They were dissolved in dimethyl sulfoxide (DMSO) and stored in the dark at −80 °C. The final concentration of DMSO or THF in the medium was 0.1% or less throughout the experiments. 

### 2.2. Cell Cultures

MC3T3-E1 cells were cultured in α-modified minimum essential medium (α-MEM; Gibco, Grand Island, NY, USA) supplemented with 10% fetal bovine serum (FBS). All cultures were maintained at 37 °C in a humidified atmosphere at 5% CO_2_ in air. When cells were treated with reagents, activated charcoal pre-treated FBS was used.

### 2.3. Measurement of Alkaline Phosphatase Activity in MC3T3-E1 Cells

After preincubation in medium supplemented 10% FBS for 24 h, MC3T3-E1 cells were treated with isoflavones and β-carotene for nine days in medium supplemented with 10% charcoal-stripped FBS. The medium was changed every three days. After incubation, cells were washed with saline, then suspended in saline containing 1% Nonidet P-40 and sonicated for 30 s. The homogenate was centrifuged at 1000 × g for 15 min, and the supernatant was used to assay for alkaline phosphatase (ALP) activity using p-nitrophenyl phosphate as the substrate. One unit of enzyme activity was defined as the activity to hydrolyze 1 nmol of substrate per milligram of protein per minute (pNPP (nmol)/protein (mg)/min).

### 2.4. Cell Growth of Preosteoblasts

To assess cell growth, MC3T3-E1 cells were seeded into 96-well plates and incubated for 24 h. The medium was then replaced with fresh medium containing 10% charcoal-stripped FBS and reagents. On day three, cells were washed with phosphate-buffered saline and assayed using the MTS (3-(4,5-dimethylthiazol-2-yl-5-(3-carboxymethoxyphenyl)-2-(4-sulfophenyl)-2H-tetrazolium) assay according to the manufacturer’s instructions (Promega, Madison, WI, USA).

### 2.5. Total RNA Isolation and Reverse Transcription Quantitative Real-Time PCR (RT-qPCR)

Total RNA was isolated from MC3T3-E1 cells using Isogen II (Nipongene, Tokyo, Japan) according to the manufacturer’s instruction. The complementary DNA was synthesized from 1 μg of total RNA using Prime Script RT Master Mix (Takara, Shiga, Japan). cDNA was quantified by real-time PCR using SYBR Premix Ex Taq II (Takara, Shiga, Japan). The PCR conditions were 95 °C for 30 s, followed by 40 cycles of 95 °C for 5 s and 60 °C for 30 s. The primer sequences used in PCR were as follows; 36B4 forward, 5′-GGCCCTGCACTCTCGCTTTC-3′; 36B4 reverse, 5′-TGCCAGGACGCGCTTGT-3′; Runx2 forward, 5′-GCAGTTCCCAAGCATTTCAT-3′; Runx2 reverse, 5′-GAAGGGTCCACTCTGGCTTT-3′; osterix forward, 5′-CCCTTCTCAAGCACCAATGG-3′; osterix reverse, 5′-AGGGTGGGTAGTCATTTGCATAG-3′; ALP forward, 5′-ACACCTTGACTGTGGTTACTGCTGA-3′; ALP reverse, 5′-CTTGTAGCCAGGCCCGTTA-3′; osteopontin forward, 5′-TGCACCCAGATCCTATAGCC-3′; osteopontin reverse, 5′-CTCCATCGTCATCATCATCG-3′ [15,16,17]. Results are expressed as the fold increase relative to the controls after normalizing to 36B4 gene expression levels.

### 2.6. Statistical Analysis

All experiments were repeated in triplicate and representative experiments are shown in the figures. Data are expressed as means ± standard error of the mean (SEM). Statistical analysis was performed using one- or two-way analysis of variance (ANOVA). Where differences were significant by one-way ANOVA, groups were compared using Tukey’s test. *p*-values < 0.05 were considered to be significant differences. Statistical analysis was performed using SPSS version 15 (IBM, Chicago, IL, USA).

## 3. Results

### 3.1. Effect of Soy Isoflavones and β-Carotene on ALP Activity in MC3T3-E1 Cells

**Figure 1:**
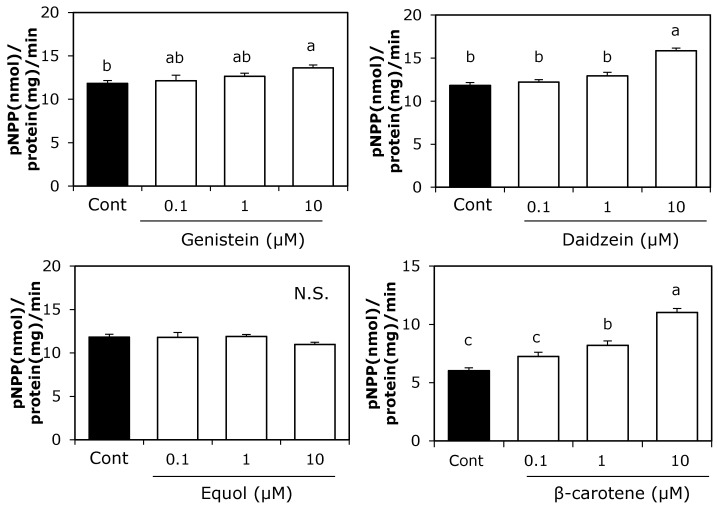
Effect of individual treatment with soy isoflavones or β-carotene on ALP activity in preosteoblast MC3T3-E1 cells. Cells were precultured in α-MEM/10% FBS for 24 h and subsequently cultured in medium with 10% charcoal-treated FBS containing 0.1–10 μM of each of the soy isoflavones or β-carotene for nine days. Data are expressed as means ± SEM (*n* = 4). Different letters indicate significant differences (*p <* 0.05). N.S.: not significant.

**Figure 2:**
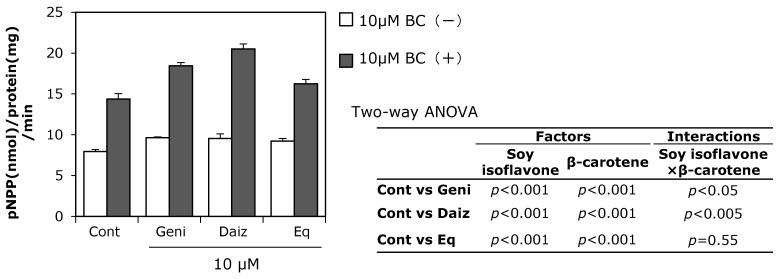
Combined effect of soy isoflavones with β-carotene on ALP activity. MC3T3-E1 cells were precultured in α-MEM/10% FBS for 24 h and subsequently cultured in medium with 10% charcoal-treated FBS containing 10 μM of soy isoflavones with (black bars) or without (open bars) 10 μM of β-carotene (BC) for nine days. Data are expressed as means ± SEM (*n* = 4). Statistical analysis was performed using two-way ANOVA with the two factors being soy isoflavones (control: Cont., daidzein: Daiz., genistein: Geni., and equol: Eq.) and β-carotene (BC). Significance was set at *p <* 0.05.

We first examined the individual effects of 0.1–10 μM soy isoflavones or β-carotene on ALP activity, which is an osteoblast differentiation marker in MC3T3-E1 cells (Figure 1). Ten μM genistein or daidzein alone significantly increased ALP activity, whereas β-carotene at both 1 and 10 μM significantly enhanced ALP activity. Soy isoflavones combined with β-carotene at 10 μM concentrations resulted in significantly higher ALP activity compared with the control. Two-way ANOVA revealed significant main effects of all soy isoflavones and β-carotene on ALP activity (Figure 2), while the interactions between two factors (daidzein and β-carotene, genistein and β-carotene) on ALP activity were also significant (*p* < 0.05).

### 3.2. Combined Effect of Soy Isoflavones with β-Carotene on MC3T3-E1 Cell Growth

**Figure 3:**
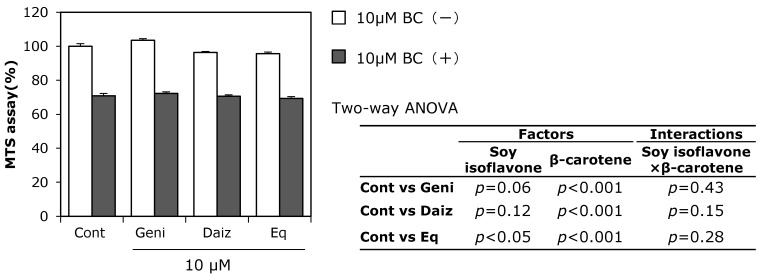
Combined effect of soy isoflavones with β-carotene on cell growth. MC3T3-E1 cells were precultured in α-MEM with 10% FBS for 24 h and subsequently cultured in medium with 10% charcoal-treated FBS containing 10 μM of soy isoflavones with (black bars) or without (open bars) 10 μM of β-carotene for three days. Cell growth was evaluated using the MTS assay. Data are expressed as means ± SEM (*n* = 6). Statistical analysis was performed using two-way ANOVA with the two factors being soy isoflavones (control: Cont., daidzein: Daiz., genistein: Geni., and equol: Eq.) and β-carotene (BC). Significance was determined at *p <* 0.05.

The combined effect of soy isoflavones and β-carotene on cell growth was examined using the MTS assay (Figure 3). Soy isoflavones and β-carotene were used at a concentration of 10 μM, as this concentration was found to have a synergistic effect on ALP activity according to two-way ANOVA. After preincubation for 24 h, MC3T3-E1 cells were treated with soy isoflavones and β-carotene for three days. Genistein and daidzein did not significantly affect cell growth, while equol and β-carotene suppressed growth. Synergistic interactions for cell growth between soy isoflavones genistein, daidzein, or equol and β-carotene, were not observed using MC3T3-E1 cells.

### 3.3. Combined Effects of Soy Isoflavones and β-Carotene on Expression of Osteoblast-Related Genes in MC3T3-E1 Cells

**Figure 4:**
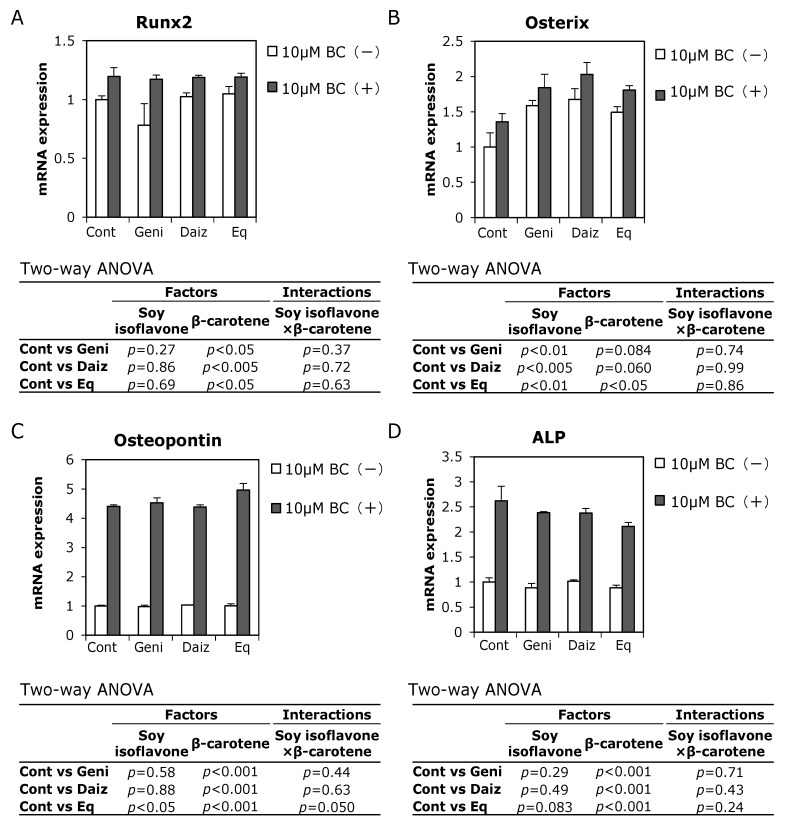
Combined effect of soy isoflavones with β-carotene on osteoblastogenic-related mRNA expression. MC3T3-E1 cells were precultured in α-MEM/10% FBS for 24 h and subsequently cultured in medium with 10% charcoal-treated FBS containing 10 μM soy isoflavones with (black bars) or without (open bars) 10 μM β-carotene (BC) for one day. Figures show the ratio of Runx2 (**A**), osterix (**B**), osteopontin (**C**), and ALP (**D**). mRNA levels are relative to control without β-carotene. Data are expressed as means ± SEM (*n* = 3). Statistical analysis was performed using two-way ANOVA with the two factors being soy isoflavones (control: Cont., daidzein: Daiz., genistein: Geni., and equol: Eq.) and β-carotene. Significance was determined at *p* < 0.05. Data are from representative experiments.

To investigate how soy isoflavones and β-carotene affect preosteoblasts, we examined their combined effect on mRNA expression levels of osteoblast differentiation-related genes in MC3T3-E1 cells. Runx2 and osterix, which are master transcription factors for controlling osteoblast differentiation, are known to regulate the expression of the osteoblastogenic markers, osteopontin and ALP [18]. Beta-carotene increased the expression of Runx2 mRNA in MC3T3-E1 cells, but soy isoflavones did not change their expression levels (Figure 4A). Conversely, all soy isoflavones significantly increased the expression of osterix mRNA, with β-carotene tending to increase osterix mRNA expression (Figure 4B).

Osteopontin and ALP are early markers of osteoblast differentiation [19], representing an osteoblast matrix protein and mineralization-related enzyme, respectively. Expression levels of both osteopontin and ALP mRNA were significantly increased following combination treatment with 10 μM β-carotene and each soy isoflavone compared to treatment with soy isoflavone alone in MC3T3-E1 cells on day one (Figure 4C,D). In addition, two-way ANOVA detected a significant main effect of β-carotene but not of soy isoflavone on the expression of these markers, with the exception of equol on osteopontin expression.

When used in combination, the interactions between soy isoflavones (daidzein, genistein, and equol) and β-carotene were not significant for each of these experiments. Therefore, the combination of soy isoflavones with β-carotene had an additive effect on promoting mRNA expression levels of osteoblast differentiation-related genes.

### 3.4. Effect of RAR Pan-Antagonist on Osteoblast-Related Gene Expression Induced by Treatment with β-Carotene

**Figure 5:**
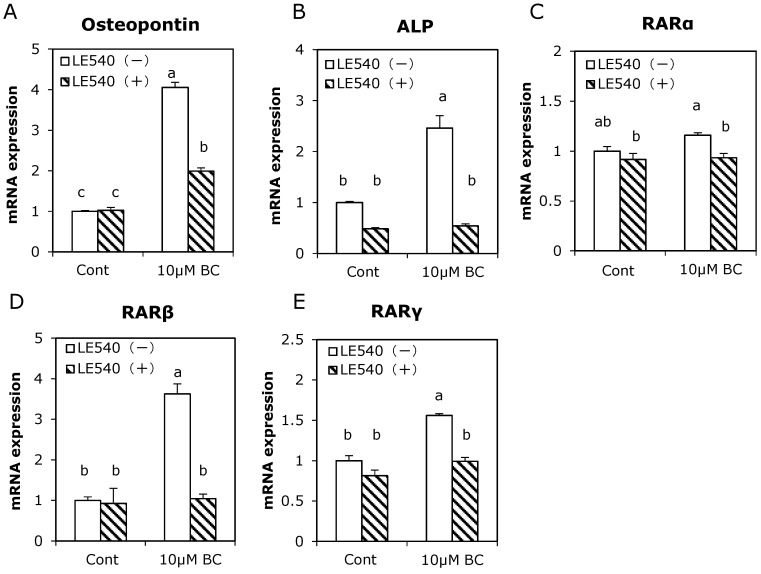
Effect of RAR antagonist on enhanced osteoblast-related gene expression with β-carotene in MC3T3-E1 cells. Cells were precultured in α-MEM/10% FBS for 24 h and subsequently cultured in medium with 10% charcoal-treated FBS containing 10 μM of β-carotene (BC) with (hatched bars) or without (open bars) 10 μM of RAR antagonist LE540 for one day. Figure shows the ratio of osteopontin (**A**), ALP (**B**), RARα (**C**), RARβ (**D**), and RARγ (**E**) mRNA levels relative to control without LE540. Data are expressed as means ± SEM (*n* = 3). Different letters indicate significant differences (*p <* 0.05). Data are from representative experiments.

The metabolite of β-carotene is known to bind to RARs and induce the differentiation of a variety of cells [20]. To investigate whether β-carotene acted in MC3T3-E1 cells via RARs, cells were treated with the RAR pan-antagonist LE540 in the presence of β-carotene (Figure 5). The osteopontin and ALP mRNA expression levels that increased following treatment with β-carotene were significantly suppressed following treatment with LE540. In addition, although treatment with β-carotene enhanced RARβ and RARγ mRNA expression, high expression levels of these mRNAs were inhibited by LE540.

## 4. Discussion

In this study, the combined effects of soy isoflavones and β-carotene on osteoblast differentiation were investigated. Although daidzein and genistein did not affect cell growth, they increased ALP activity in MC3T3-E1 cells, which is consistent with previous studies [13,21]. Soy isoflavones have a structural similarity to estrogen and weak affinity for estrogen receptors [22]. Similar to estrogen, soy isoflavones are reported to promote osteoblast differentiation via ERs [23,24]. In addition, estrogen enhances osteoblast proliferation and collagen gene expression [25]. These reports suggest that soy isoflavones are useful in maintaining bone formation via estrogen-like effects. In this study, equol alone did not significantly increase ALP activity (Figure 1). Equol, a daidzein metabolite, is a chiral molecule, which exists as enantiomers R-equol and S-equol. Setchell *et al.* [26] report that S-equol, but not R-equol, has a relatively high affinity for ERs. Furthermore, Wang *et al.* [27] report that equol enhances ALP activity via ERs in primary osteoblasts. Because we used the racemic form of equol, the effect of equol on osteoblast differentiation may be attenuated by its form. 

Beta-carotene markedly enhanced ALP activity with inhibition of cell growth (Figure 1 and Figure 3). Park *et al.* [12] report that the cell growth inhibition induced by treatment with β-carotene in MC3T3-E1 cells results from suppression of DNA synthesis, which is often observed during cell differentiation. In this study, the concentration of β-carotene that induced cell growth inhibition was similar to the concentration for causing enhanced ALP activity, suggesting that the inhibition effect of β-carotene on cell growth might be related to cell differentiation.

It has been shown that a higher serum concentration of β-carotene is involved in the prevention of bone loss in post-menopausal Japanese women [10]. *In vitro*, β-carotene and its metabolites retinol and retinoic acid are reported to induce osteoblast differentiation [12]. The ALP gene has an RAR binding site called the retinoic acid receptor element within its promoter region [28]. In addition, the RAR activator induces early osteoblastic markers in pluripotent mesenchymal cells [29]. In this study, the RAR inhibitor suppressed changes in ALP, osteopontin, RARβ, and RARγ mRNA expression induced by treatment with β-carotene (Figure 5). Therefore, β-carotene may enhance osteoblast differentiation, at least in part via RAR signaling. Conversely, retinoic acid is reported to suppress mature osteoblast differentiation [30]. Although we treated pre-osteoblast MC3T3-E1 cells with β-carotene for nine days, the stimulatory effect of 10 μM β-carotene on ALP activity was considerably weaker than that of 10 μM retinoic acid (date not shown). The effects of β-carotene or vitamin A might differ at each stage of osteoblast differentiation.

The combination of soy isoflavones with β-carotene exhibited higher ALP activity than treatment with isoflavones alone. Additionally, these food components had an interaction on ALP activity according to two-way ANOVA, suggesting the effect was synergistic. However, although there was a significant main effect of β-carotene on enhanced Runx2, osteopontin, and ALP mRNA expression, soy isoflavones had no significant effect on the expression of these mRNAs one day after treatment. A significant main effect of soy isoflavones, but not β-carotene, on osterix mRNA expression was observed. The interactions between soy isoflavones and β-carotene were therefore not significant on osteoblast differentiation-related gene expression. These results together with ALP activity raise the possibility that each soy isoflavone and β-carotene affect osteoblast differentiation via different pathways, such as ERs or the RAR signaling pathway, respectively. 

Previously, we reported that a combination of soy isoflavones and caroteonoids, including β-carotene, prevented osteoclast formation *in vitro* [14]. In this study, the combination of soy isoflavones with β-carotene was shown to enhance early osteoblastic differentiation. These results suggest that intake of a combination of soy isoflavones and β-carotene might be useful for maintaining bone health. 

## 5. Conclusions

This study demonstrated combined effects of soy isoflavones and β-carotene on differentiation of osteoblastic cells. Soy isoflavones combined with β-carotene resulted in higher ALP activity than treatment with isoflavone or β-carotene alone. However, each of the soy isoflavones and β-carotene affected different osteoblast-related genes at the early stage of differentiation, and the interactions between the components were not significant. These results suggest that combination intakes of soy isoflavones and β-carotene may be useful for maintaining a positive balance of bone turnover by inducing osteoblast differentiation. However, further studies are required to confirm these effects *in vivo*.
